# Supplementary material for: Preparation of TiO2 Nanorods@Ni-Foam for Photocatalytic Decomposition of Acetaldehyde—In Situ FTIR Surface Investigation
Source: Materials (Basel). 2025 Feb 24;18(5):986. doi: 10.3390/ma18050986 (PMC11901285; doi:10.3390/ma18050986)
Supplement: Supplementary file 1 [file materials-18-00986-s001.zip › materials-3471376-supplementary.pdf]

Supplementary material for:

Preparation of TiO_2 nanorods@Ni-foam for photocatalytic decomposition of acetaldehyde – in situ FTIR surface investigation

Piotr Rychtowski *, Bartłomiej Prowans, Piotr Miądlicki, Maciej Trzeciak and Beata Tryba *

Department of Catalytic and Sorbent Materials Engineering, Faculty of Chemical Technology and Engineering, West Pomeranian University of Technology in Szczecin, Pułaskiego 10, 70-322 Szczecin, Poland; bartlomiej.prowans@zut.edu.pl (B.P.); piotr.miadlicki@zut.edu.pl (P.M.); maciej.trzeciak@zut.edu.pl (M.T.)

* Correspondence: piotr.rychtowski@zut.edu.pl (P.R.); beata.tryba@zut.edu.pl (B.T.)

Table S1. Phase composition summary of the studied samples determined from the Rietveld refinement

Phase (%) / Sample	Anatase	Brookite	Rutile	$Na_2Ti_3O_7$
A-TNR	–	4.1	19.2	76.7
A-TNR-0	–	3.6	26.1	70.3
A-TNR-5	–	–	17.8	82.2
A-TNR-10	76.1	22.0	1.4	0.5
A-TNR-15	80.9	18.5	–	0.6
A/R-TNR	–	–	21.1	78.9
A/R-TNR-0	–	6.3	25.6	68.0
A/R-TNR-5	17.5	21.3	1.0	60.2
A/R-TNR-10	58.2	25.6	13.3	2.9
A/R-TNR-15	–	–	20.7	79.3

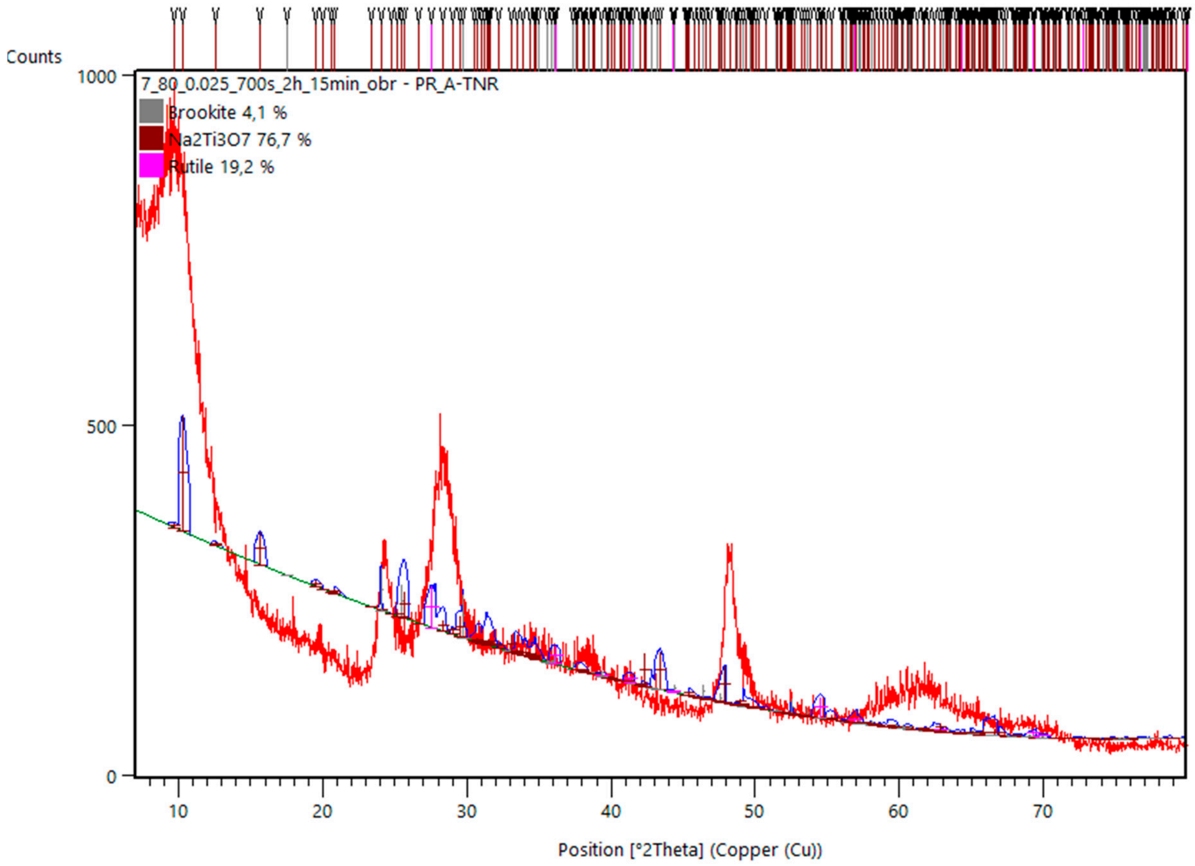

Figure S1. Rietveld refinement of XRD diffractogram of sample originated from anatase and before second hydrothermal step (A-TNR)

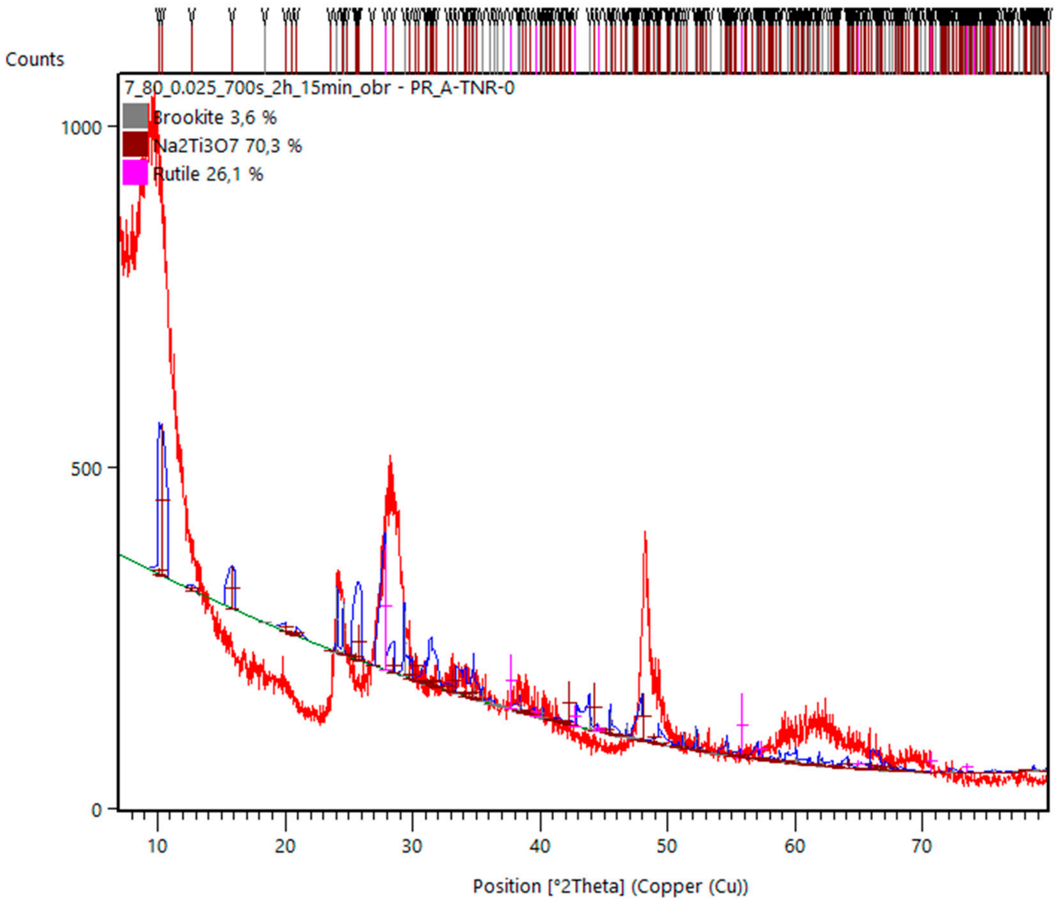

Figure S2. Rietveld refinement of XRD diffractogram of sample originated from anatase and untreated by the nitric acid (A-TNR-0)

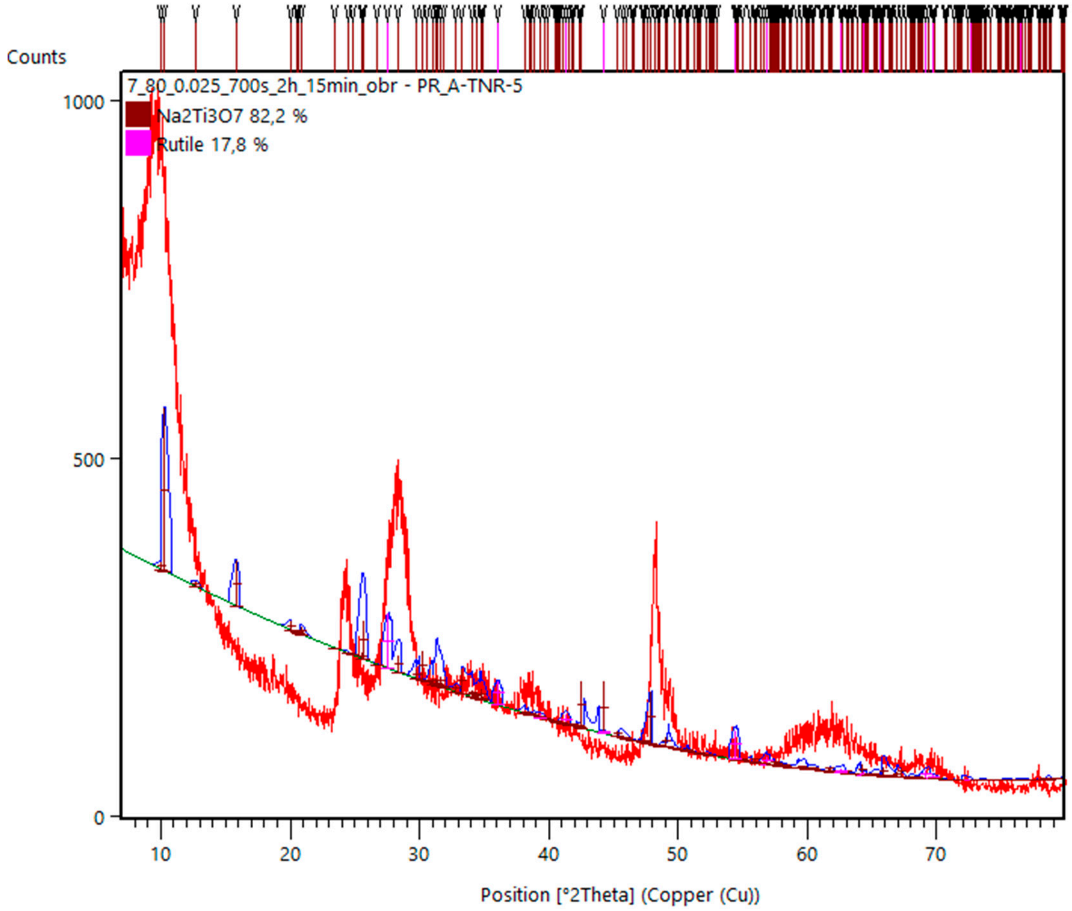

Figure S3. Rietveld refinement of XRD diffractogram of sample originated from anatase and treated by 5 ml of nitric acid (A-TNR-5)

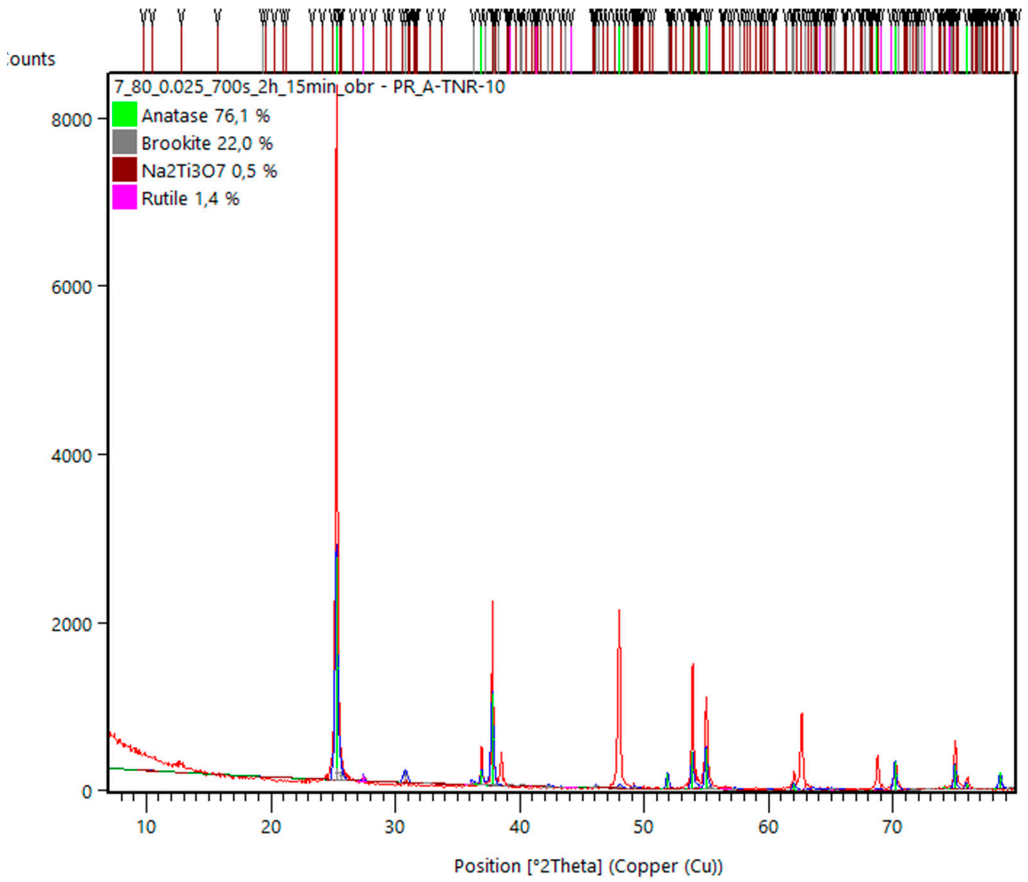

Figure S4. Rietveld refinement of XRD diffractogram of sample originated from anatase and treated by 10 ml of nitric acid (A-TNR-10)

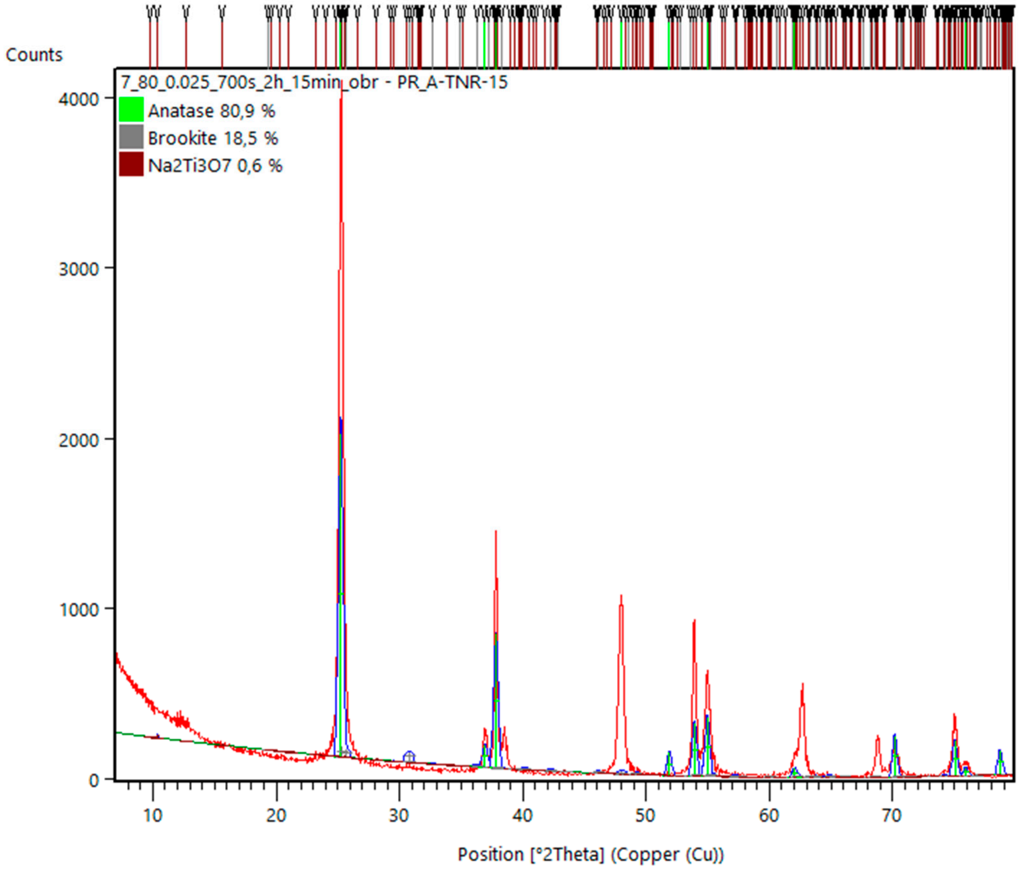

Figure S5. Rietveld refinement of XRD diffractogram of sample originated from anatase and treated by 15 ml of nitric acid (A-TNR-15)

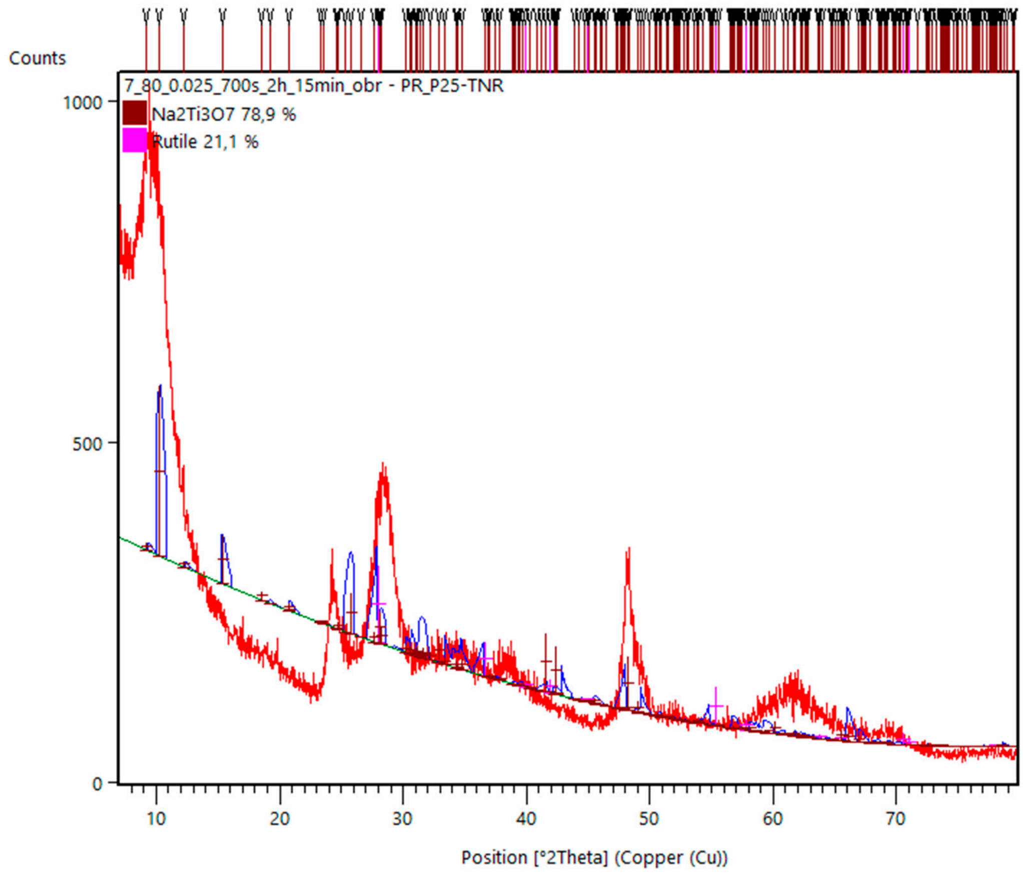

Figure S6. Rietveld refinement of XRD diffractogram of sample originated from the mixture of anatase and rutile, before second hydrothermal step (A/R-TNR)

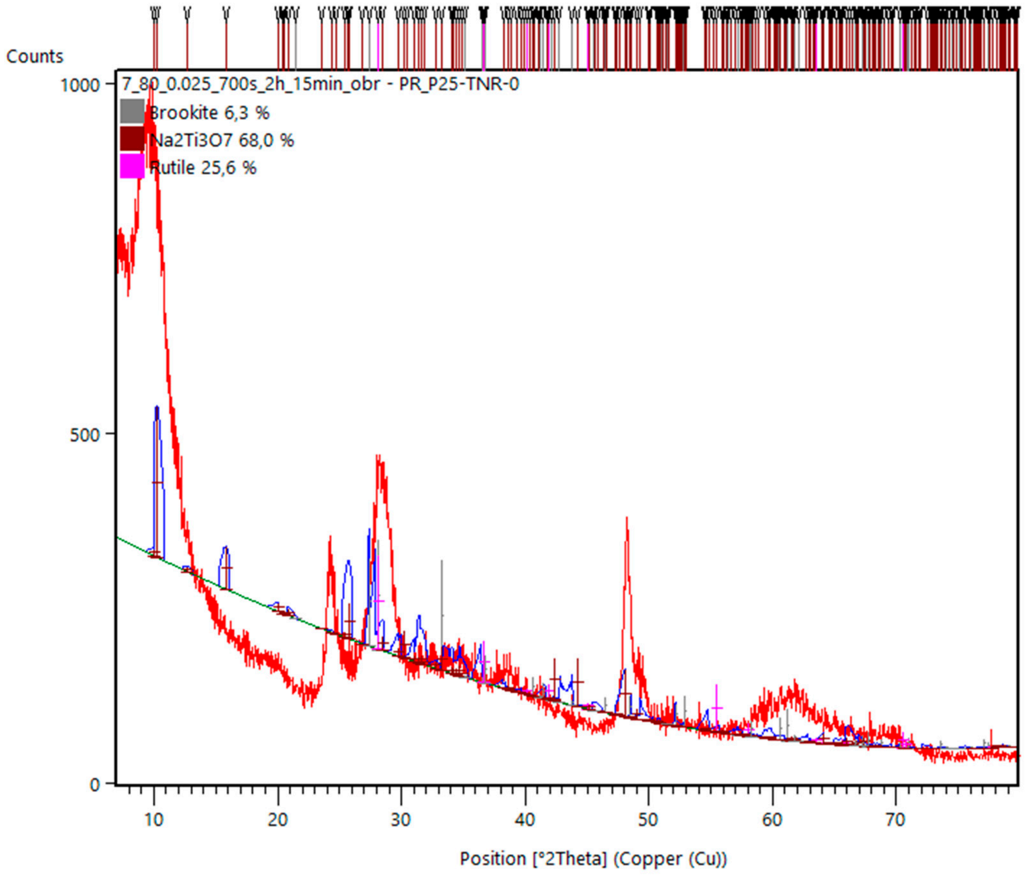

Figure S7. Rietveld refinement of XRD diffractogram of sample originated from the mixture of anatase and rutile, untreated by the nitric acid (A/R-TNR-0)

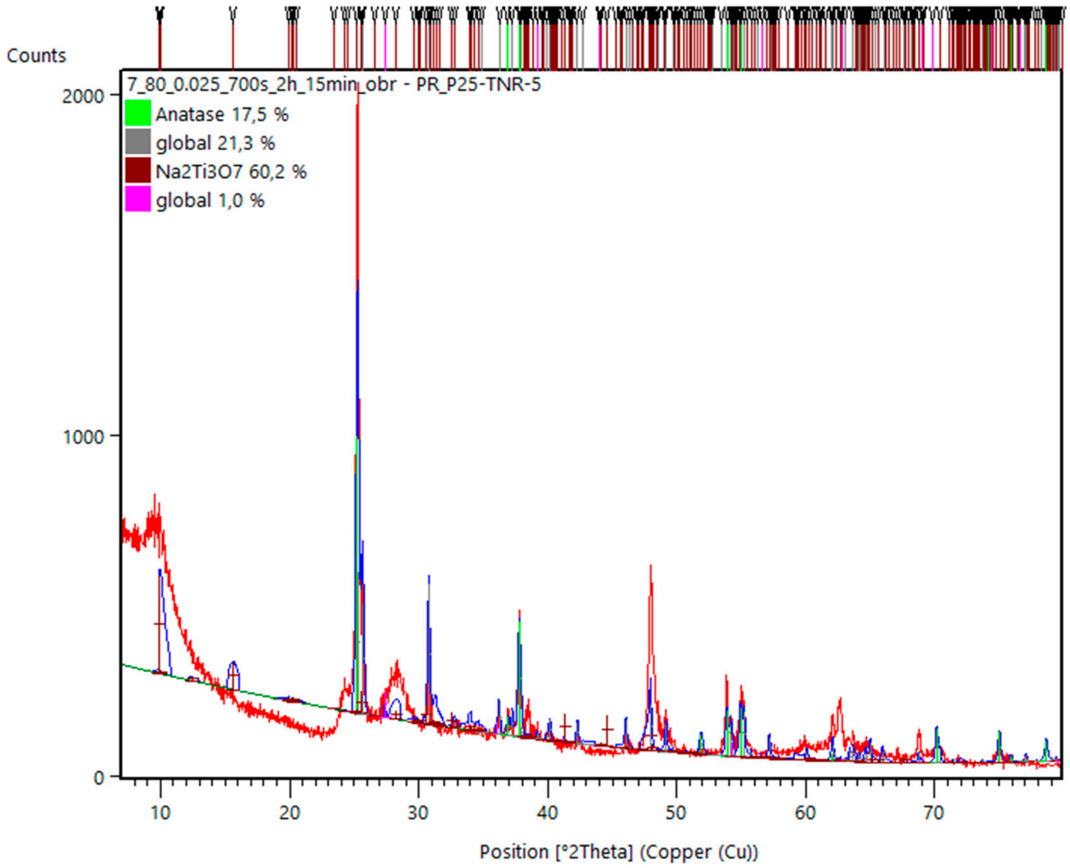

Figure S8. Rietveld refinement of XRD diffractogram of sample originated from the mixture of anatase and rutile, treated by 5 ml of nitric acid (A/R-TNR-5)

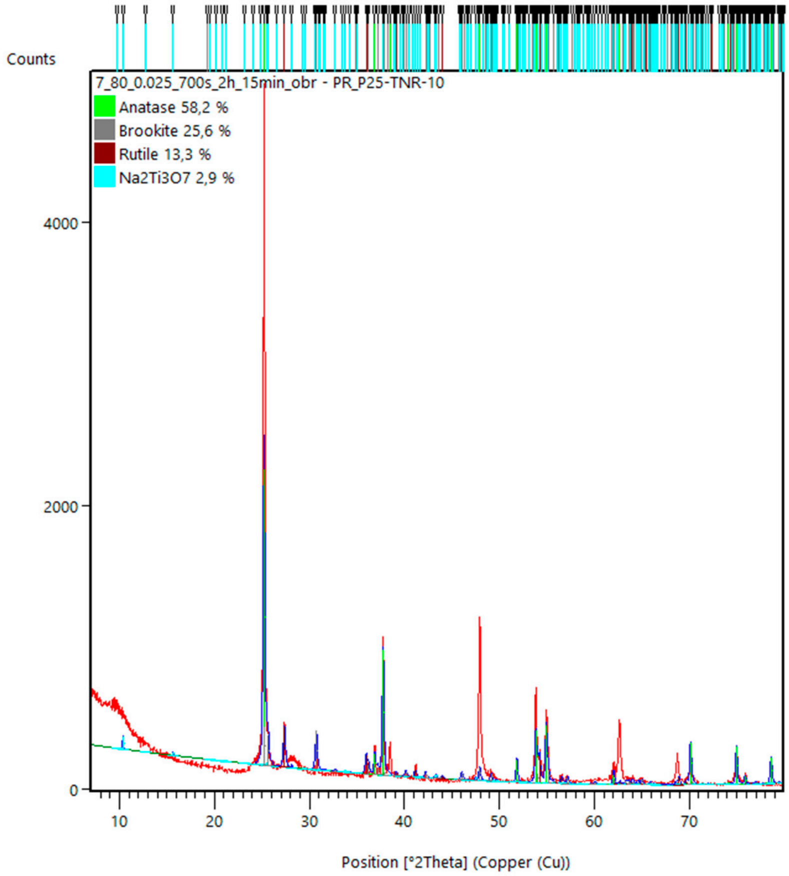

Figure S9. Rietveld refinement of XRD diffractogram of sample originated from the mixture of anatase and rutile, treated by 10 ml of nitric acid (A/R-TNR-10)

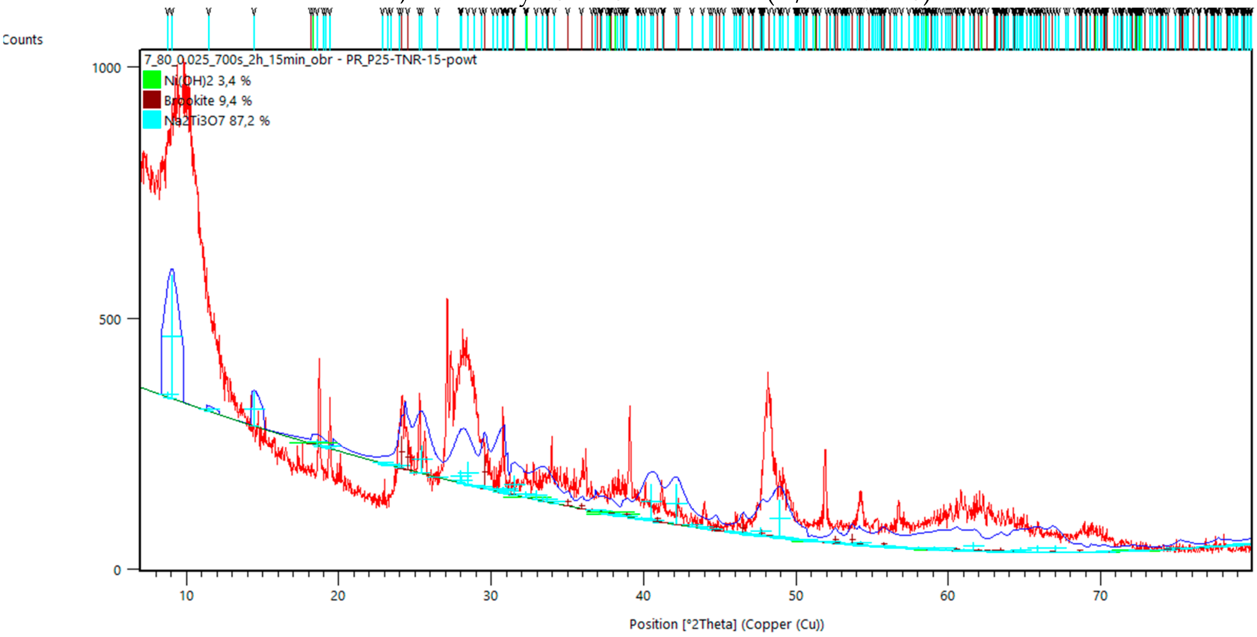

Figure S10. Rietveld refinement of XRD diffractogram of sample originated from the mixture of anatase and rutile, treated by 15 ml of nitric acid (A/R-TNR-15)
